# Long‐Term Outcomes From a Randomized Controlled Trial of Acceptance and Commitment Therapy (ACT) Compared to Standard Medical Care for Improving Quality of Life in Muscle Disorders

**DOI:** 10.1002/mus.28322

**Published:** 2024-12-29

**Authors:** Christopher D. Graham, Michael Rose, Victoria Edwards, Chiara Vari, Nicola O'Connell, Emma Taylor, Lance M. McCracken, Aleksander Radunovic, Wojtek Rakowicz, Sam Norton, Trudie Chalder

**Affiliations:** ^1^ Department of Psychological Sciences & Health, Graham Hills Building University of Strathclyde Glasgow Scotland; ^2^ Department of Neurology King's College Hospital London UK; ^3^ Department of Psychological Medicine Institute of Psychiatry, Psychology and Neuroscience, King's College London London UK; ^4^ Department of Psychology Uppsala University Uppsala Sweden; ^5^ Barts and the London MND Centre Royal London Hospital London UK; ^6^ Wessex Neurological Service University Hospital Southampton Southampton UK; ^7^ Department of Psychology Institute of Psychiatry, Psychology and Neuroscience, King's College London London UK; ^8^ Centre for Rheumatic Disease, Department of Inflammation Biology, Faculty of Life Sciences and Medicine King's College London London UK

## Abstract

**Introduction/Aims:**

A previous randomized controlled trial showed that guided self‐help acceptance and commitment therapy plus standard medical care (ACT+SMC) was superior to standard medical care alone (SMC) for improving quality of life (QoL) and mood at 9‐weeks post randomization in a sample of people with muscle disorders (MD). This follow‐up study evaluated whether these effects were maintained in the longer term alongside individual patterns of response.

**Methods:**

The original study was a two‐arm parallel group randomized controlled trial, which compared ACT+SMC to SMC. The primary outcome of QoL was assessed with the Individualized Neuromuscular Quality of Life Questionnaire. We recruited people with different MDs from UK National Health Service clinics and patient registries. In this follow‐up study, we re‐administered all outcome measures to participants at 6 months post randomization.

**Results:**

Questionnaires were completed by 109 participants (70.3% of the original sample). At six months, the adjusted group difference in QoL continued to favor ACT+SMC, which was significant with moderate effect size. Improvements in secondary outcomes of mood and aspects of psychological flexibility also favored ACT+SMC. Reliable improvement was evident in 33.9% of the ACT+SMC group and 5.7% of the SMC group. Reliable deterioration was uncommon following ACT+SMC (1.8% of participants.)

**Discussion:**

The beneficial impacts of guided self‐help ACT for QoL and mood were maintained in the longer‐term. A third of participants showed response to this brief intervention, and negative individual outcomes were very rare. As is common in psychological interventions, there was a considerable group of non‐responders.

## 1 Introduction

Muscle disorders (MD)—such as facioscapulohumeral muscular dystrophy, limb‐girdle muscular dystrophy, inclusion body myositis, and Becker muscular dystrophy—cause muscle weakness and wasting, leading to a decline in mobility and other symptoms like fatigue and pain. Few disease modifying therapies exist, so treatment generally aims to enhance functioning via occupational therapy and physiotherapy [1]. People living with MD identify methods for improving quality of life (QoL) as a research priority [2]. Observational studies show that psychological factors, such as coping methods, illness perceptions, and psychological flexibility all contribute to QoL in MD [3, 4, 5], suggesting that psychological interventions could help improve QoL [6]. Therefore, we developed a psychological intervention for improving QoL in MD and tested this in a randomized controlled trial [7, 8, 9].

To facilitate engagement for those with impaired mobility, fatigue, or in employment, we designed a psychological intervention that was delivered remotely. We used acceptance and commitment therapy (ACT) because this approach is suited to the challenges of living with a long‐term health condition [10]. ACT uses a range of therapy methods [11] – including mindfulness, perspective‐taking exercises and goal setting—to help participants develop a quality within their behavior called psychological flexibility. This is defined as: “the capacity to persist or to change behaviour in a way that (1) includes conscious and open contact with thoughts and feelings (openness), (2) appreciates what the situation affords (awareness), and (3) serves one's goals and values (engagement)” [12].

In the two‐arm parallel groups randomized controlled trial, we randomized 155 people with MD to ACT‐based guided self‐help plus standard medical care (ACT+SMC) or standard medical care alone (SMC) [7]. At our primary end‐point of 9 weeks, those randomized to ACT+SMC reported significantly better QoL, with a moderate‐to‐large effect size. The majority of secondary outcomes (e.g., mood, functional impairment) were also significantly better in the ACT+SMC group.

While these short‐term outcomes are promising, such effects could be transient. We have not yet examined whether differences seen at 9 weeks were sustained. Neither have we investigated individual patterns of change across treatment, which can give additional useful information regarding the proportions of participants showing improvement or deterioration in outcomes during the period of study [13]. Therefore, we undertook a longer term follow‐up study and additional analyses to examine: (1) the long‐term (6 month) efficacy of our ACT‐based guided self‐help intervention by comparing ACT+SMC and SMC arms on primary and secondary outcomes and (2) the pattern of response to treatment by assessing change in individual outcomes (reliable change) across the period of study in both arms.

## 2 Methods

### 2.1 Design

The primary study [7, 8], a two‐arm parallel groups randomized controlled trial that compared ACT guided self‐help intervention plus standard medical care (ACT+SMC) to SMC alone, was pre‐registered at ClinicalTrials.gov (Identifier: NCT02810028.) This trial recruited adults with MD from UK National Health Service clinics and patient registries. The primary outcome was QoL, and secondary outcomes included mood, functioning, and psychological flexibility; all were recorded with standardized questionnaires. Outcomes for the main part of this trial were recorded at 3, 6, and 9 weeks.

Ethical approval was provided by the London‐Camberwell St Giles Research Ethics Committee (16/LO/0609). Research governance approval was obtained from King's College Hospital NHS Foundation Trust and King's College London. Participants gave informed consent prior to initiation of trial procedures.

### 2.2 Participants

We included participants with one of four MDs: limb girdle muscular dystrophy, Becker muscular dystrophy, facioscapulohumeral muscular dystrophy (FSH), inclusion body myositis (IBM). These are frequent presentations to specialist MD clinics, which share key characteristics—rare disease and key symptoms (e.g., muscle wasting and weakness, pain and fatigue) – meaning that many downstream psychological challenges are common. They also do not tend to involve cognitive impairment, meaning that the intervention shouldn't require significant adaptation across participants to enable access.

Inclusion criteria were: age of 18 years and older; a diagnosis of MD for more than six months; access to the internet and a computer; scores of ≥ 8 for depression or anxiety on the Hospital Anxiety and Depression Scale (HADS). Exclusion criteria were: unstable complications of MD (e.g., respiratory weakness, cardiomyopathy); major active comorbidities unrelated to MD (e.g., cardiovascular disease, respiratory disease); a current diagnosis of an active major mental health disorder likely to interfere with participation (e.g., psychosis, disordered eating); current or recent participation in other treatment intervention studies (< 4 weeks after completion); currently receiving psychological support or psychotherapy; inability to read English questionnaires; cognitive impairment.

### 2.3 Procedure

For the primary study [7, 8], we identified and recruited participants from UK National Health Service MD clinics, MD research registries and via the charity MD‐UK. The independent randomization service at the King's Clinical Trial Unit randomized participants to arm at the individual level via block randomization with randomly varying block sizes stratified by recruiting site.

The pre‐registered timings of outcome measurement were at 3‐, 6‐, and 9‐weeks post‐randomization. The additional long‐term follow‐up time point was at 6 months post‐randomization. Baseline measures were collected by research assistants face‐to‐face in NHS clinics or on the telephone. The 3, 6, and 9 week and 6 month outcomes were collected online using the Online Surveys programme, via a link e‐mailed to participants.

### 2.4 Guided Self‐Help ACT

The guided self‐help ACT intervention is described in the published protocol [8]. This consisted of four modules of written material and audio files. The modules were supported by five 15–30 min telephone support sessions with a clinical psychologist. The modules contained commonly used ACT exercises, which were presented to participants as a series of four psychological skills. Skill 1, Mindfulness, consisted of practice of brief centering and willingness exercises; Skill 2, Unhooking, involved diffusion/verbal distancing exercises; Skill 3, Follow your values, encouraged participants to identify and undertake activity consistent with their over‐arching values; Skill 4, Take an observer perspective, encouraged flexible perspective‐taking. The telephone support sessions aimed to help participants apply the skills in their everyday life.

### 2.5 Standard Medical Care (SMC)

For the entirety of the trial, all participants had access to SMC, in line with current medical practice. This consisted of periodic review of functional impairment arising from muscle weakness and corresponding suggestions to reduce associated disability via the use of assistive devices or adaptations to housing. Medical professionals answered queries, provided information produced by charities on the condition, and sign‐posted to local support groups. Participants also had access to local physiotherapy input. At the time of trial, it was unusual for sites to offer routine review with a mental health professional.

### 2.6 Primary Outcome Measure

The primary outcome was QoL, measured with the Individualized Neuromuscular Quality of Life Questionnaire (INQoL), Life Area domain. The INQoL ‐ a QOL measure designed specifically for MD ‐ has 45 items within 10 domains, capturing the impact of MD symptoms on QoL. The Life Area domain is a sub‐section of the overall measure that is calculated by averaging five subscales measuring the impact of MD on activities (5 items), independence (3 items), social functioning (10 items), emotional functioning (6 items), and body image (3 items) [14]. Scores can range from 0–100, and a higher score is indicative of worse QoL. The INQoL shows acceptable psychometric properties, with Cronbach's alphas above 0.70 across each life area domain [15].

### 2.7 Secondary Outcomes

We used a range of secondary outcomes that were assessed at all timepoints. Mood was measured with the Hospital Anxiety and Depression Scale (HADS) [16]. This has 14 items: 7 pertain to anxiety; 7 to depression. Scores for each domain range from 0–21, with higher scores indicating more severe mood disturbance. We assessed weakness (3 items), fatigue (3 Items), and pain (3 items) subscales of the INQoL, as well as the subdomains used to generate the total score (14 items). For each, scores can range from 0–100, with higher scores indicating worse QoL. Symptom interference was measured with the 5‐item Work and Social Adjustment Scale (WSAS) [17], which generates scores ranging from 0–40, with higher scores indicative of greater symptom interference. Physical impairment was assessed with the Stanford Health Assessment Questionnaire Disability Index (HAQ‐DI) [18]—a 20‐item measure with scores that can range from 0–3, which are averaged, with higher scores indicating greater levels of physical impairment. Where indicated, the Inclusion Body Myositis (IBM) Functional Rating Scale [19] was also used to measure physical impairment—a functional rating scale with 10 points, where higher scores indicate greater physical impairment. In addition, at the 6‐month assessment only, we assessed participant perception of change with the Patient Global Impression of Change scale (PGIC) [20], which uses a 7‐point ordinal scale from ‘very much worse’ to ‘very much better’, and treatment satisfaction on a similar 7‐point scale from ‘very dissatisfied’ to ‘very satisfied’.

We also included measures corresponding to the three aspects of the psychological flexibility. The Acceptance and Action Questionnaire (AAQ‐II) [21], is the most widely used measure of psychological flexibility, largely measuring ‘openness’ processes. It has 7 items, and generates a score ranging from 7–49, with higher scores indicative of poorer psychological flexibility/openness. The Mindfulness Attention Awareness Scale (MAAS) [22] is a 15‐item measure of present moment focus, measuring ‘awareness’ processes, scores range from 1–6, with higher scores an indicator of greater present moment focus. The Committed Action Scale (CAQ) [23] was used to measure ‘engagement’ processes. This has 8 items, and produces a score of 0–48, with higher scores suggestive of greater engagement.

### 2.8 Analysis

The Consolidated Standards of Reporting Trials (CONSORT) guideline [24] was used for reporting the results of this study. The statistician was not blind to treatment group allocation for the 6‐month analysis due to this being a follow‐up study after the primary analysis had already been conducted. The previous analysis was undertaken blind to group allocation. Analyses were conducted using Stata MP 15.1 (Stata‐Corp, College Station, TX, USA.)

Long‐term (6 month) efficacy analyses of our ACT‐based guided self‐help intervention was conducted by comparing arms on primary and secondary outcomes. We used the same strategy as the original primary outcome analysis. Specifically, estimates of treatment effects at 6 months post‐randomization were based on adjusted mean differences using linear‐mixed models using an intention‐to‐treat. A two‐level model was estimated including a random intercept to account for repeated assessments within individuals over time. Covariates in the model included an indicator variable for group assignment, an indicator for follow‐up time, group by time interaction terms, the baseline level of the outcome variable, and indicator variables for the recruitment center as this was a stratification factor in the randomization. For the PGIC and treatment satisfaction, outcomes were recorded at 6 months only—treatment effects were estimated using ordinal logistic regression due to the response scale used. These included an indicator variables group assignment and recruiting center. Standardized Mean Difference was used to calculate effect sizes. We interpreted these based on the assumption that an SMD of 0.2 indicates a small effect, greater than or equal to 0.5 a medium effect and greater than or equal to 0.8 a large effect [25].

Treatment effects for the primary and most secondary outcomes were estimated as adjusted mean differences at each timepoint using a mixed‐effects models. Missing data sensitivity analyses using a pattern‐mixture model approach were reported for the primary outcome in main trial publication at 9 weeks. These were repeated but are not reported here as there was no substantive change in the interpretation from the main publication and the treatment effect on the primary outcome was stable under a plausible range of missing data values. These adjusted for baseline level of the outcome and recruiting site using the intention to treat sample, *N* = 148.

Individual response to treatment was examined by assessing change in the primary outcome at 6 months. Data were categorized based on an estimate of reliable change where this was assumed to be 1.96 times the standard error of the measurement [26]. This was estimated to be a change of 15 points or more, based on an SD of 18 and reliability of 0.9. To aid interpretation, levels of the primary outcome at 6 months were plotted against baseline level of the primary outcome by treatment group. On the primary outcome measure, a deterioration in score is assumed if scores increase to a greater extent than measurement error; an improvement is assumed if scores decrease to a greater extent than measurement error.

## 3 Results

**FIGURE 1:**
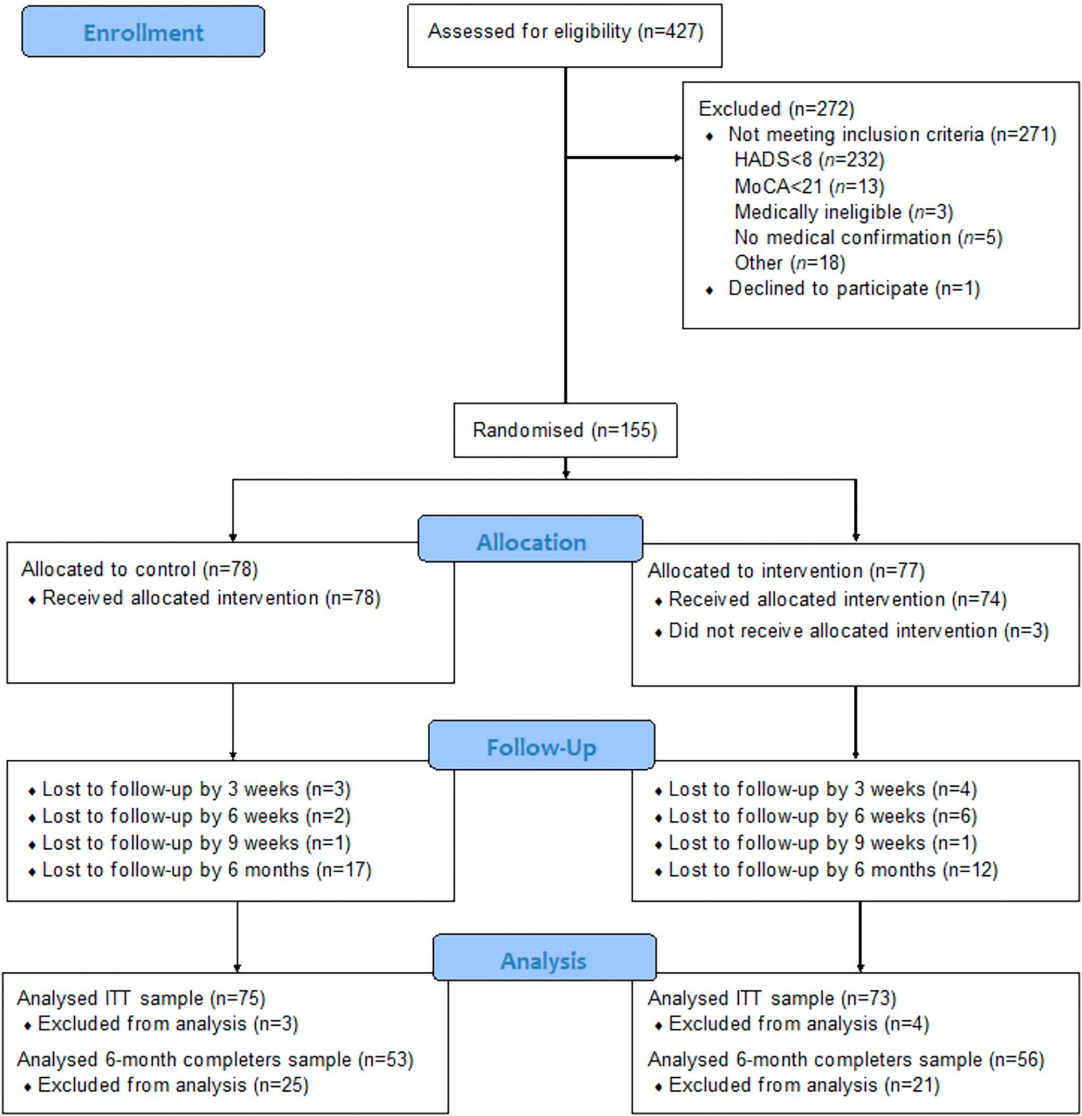
CONSORT diagram showing participant flow through all stages of the study to 6 months.

A total of 155 participants were randomized, with 109 (70.3%) providing data at 6 months (Figure 1). Due to the nature of the methods used, the 6‐month treatment effect estimates included data from all people providing data on at least one of the follow‐up assessments (*N* = 148) but the reliable change analysis was restricted to only those with complete data (*n* = 109). Facioscapulohumeral muscular dystrophy and inclusion body myositis were the most common diagnoses, and very few people with Becker muscular dystrophy participated (Table 1). Most participants were white, and the sample comprised a relatively even number of men and women. There were no significant differences between the 109 who completed the 6‐month assessment compared to those who did not on any demographic or clinical variable (Table 1).

**TABLE 1:** Demographic details on the sample included in the analyses at baseline and 6‐month follow‐up.

		Baseline		6‐month follow‐up	
		SMC	ACT+SMC	SMC	ACT+SMC
		*N* = 75	*N* = 73	*N* = 53	*N* = 56
Age (years)		54 (44–69)	51 (37–62)	55 (46–69)	51.5 (38–63.5)
Sex	Female	31 (41%)	42 (58%)	22 (42%)	32 (57%)
	Male	44 (59%)	31 (42%)	31 (58%)	24 (43%)
Ethnicity	White	68 (91%)	65 (89%)	49 (92%)	52 (93%)
	Non‐white	7 (9%)	8 (11%)	4 (8%)	4 (7%)
MD type	Limb Girdle	16 (21%)	19 (26%)	9 (17%)	14 (25%)
	Becker MD	2 (3%)	2 (3%)	0 (0%)	2 (4%)
	FSHD	36 (48%)	38 (52%)	28 (53%)	30 (54%)
	IBM	21 (28%)	14 (19%)	16 (30%)	10 (18%)
Years since diagnosis		14.5 (6–27.5)	12.5 (7–20)	12 (6–22)	12 (5–21)
INQoL Total, baseline		62 (45–69)	64 (47–75)	64 (46–69)	61.5 (45.5–73)

*Note*: Data are provided as (%) or median (IQR).

Abbreviations: FSHD = facioscapulohumeral muscular dystrophy; IBM = inclusion body myositis; INQoL = Individualized Neuromuscular Quality of Life questionnaire; MD = muscle disorder.

**FIGURE 2:**
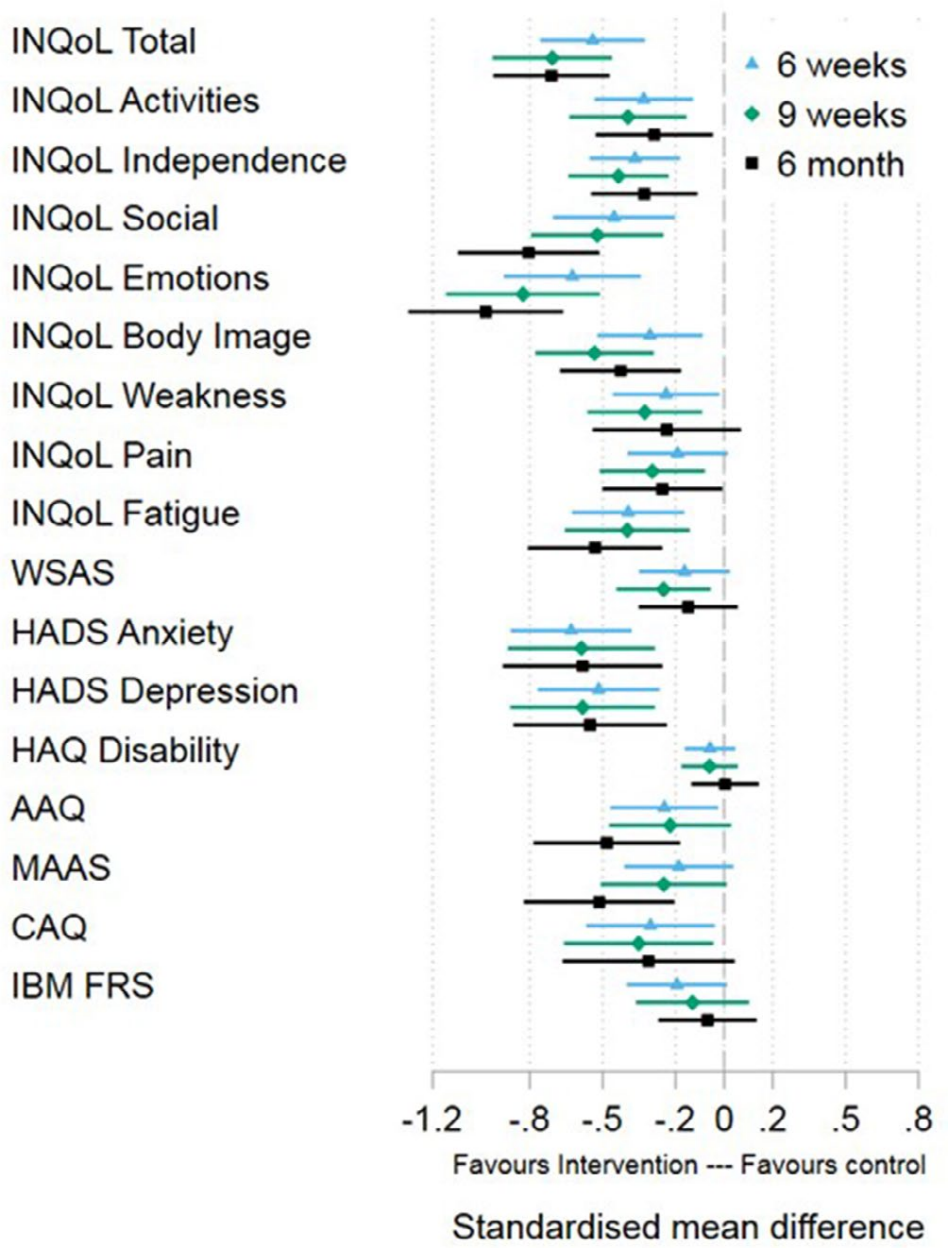
Forest plot showing standardized effect sizes for primary and secondary outcome measures at 6 weeks, 9 weeks and 6 months. AAQ = Acceptance and Action Questionnaire‐II; CAQ = Committed Action Questionnaire; HADS = Hospital Anxiety & Depression Scale; HAQ = Health Assessment Questionnaire; IBM‐FRS = Inclusion Body Myositis Functional Rating Scale; INQoL = Individualized Neuromuscular Quality of Life Questionnaire; MAAS = Mindfulness Attention Awareness Scale; WSAS = Work & Social Adjustment Scale.

Those in the ACT+SMC group showed significantly better INQoL total scores (primary outcome) at 6 months than those randomized to SMC alone (Supplementary Table 1.) Specifically, scores were 12.3 points lower (95%CI: −16.4 to −8.12), equating to a standardized mean difference of −0.71. and maintenance of the moderate to large effect size seen at 9 weeks [7] (Figure 2).

Among secondary outcomes (Figure 2, Supplementary Table 1), effects were generally maintained or improved for the social and emotional functioning subscales of the INQoL, which continued to be significantly better in the ACT+SMC group at 6 months compared to SMC alone. The small significant improvement in symptom interference (WSAS) in the ACT+SMC group, compared to SMC alone, seen at 9 weeks, was reduced and no longer significant at 6 months. As at 9 weeks, those in the ACT+SMC group did not show significant improvements in physical disability at 6 months (HAQ; IMB FRS), compared to SMC alone. Regarding variables assessing ACT processes, the openness (AAQII) and awareness (MAAS) measures of psychological flexibility were significantly better in the ACT‐SMC group at 6‐months compared to SMC alone. However, there was no statistically significant difference between groups in engagement score (CAQ).

Patient impression of change and satisfaction with treatment were assessed at 6 months only (see Supplementary Table 2). Around 32% of those who completed ACT reported impressions of improvement following ACT (i.e., selected responses options ‘a little better’ to ‘very much better’), compared to 6% of those in the SMC arm. Treatment effects on these variables, assessed using ordinal logistic regression, demonstrated a four‐fold increased odds of perceived greater improvement in health in the ACT+SMC group (OR = 4.0; 95%CI = 1.9 to 8.5); *p* < 0.001) and nine‐fold greater satisfaction (OR = 8.9; 95%CI = 3.6 to 22.1; *p* < 0.001), compared to SMC alone.

### 3.1 Patterns of Individual Change in QoL

**FIGURE 3:**
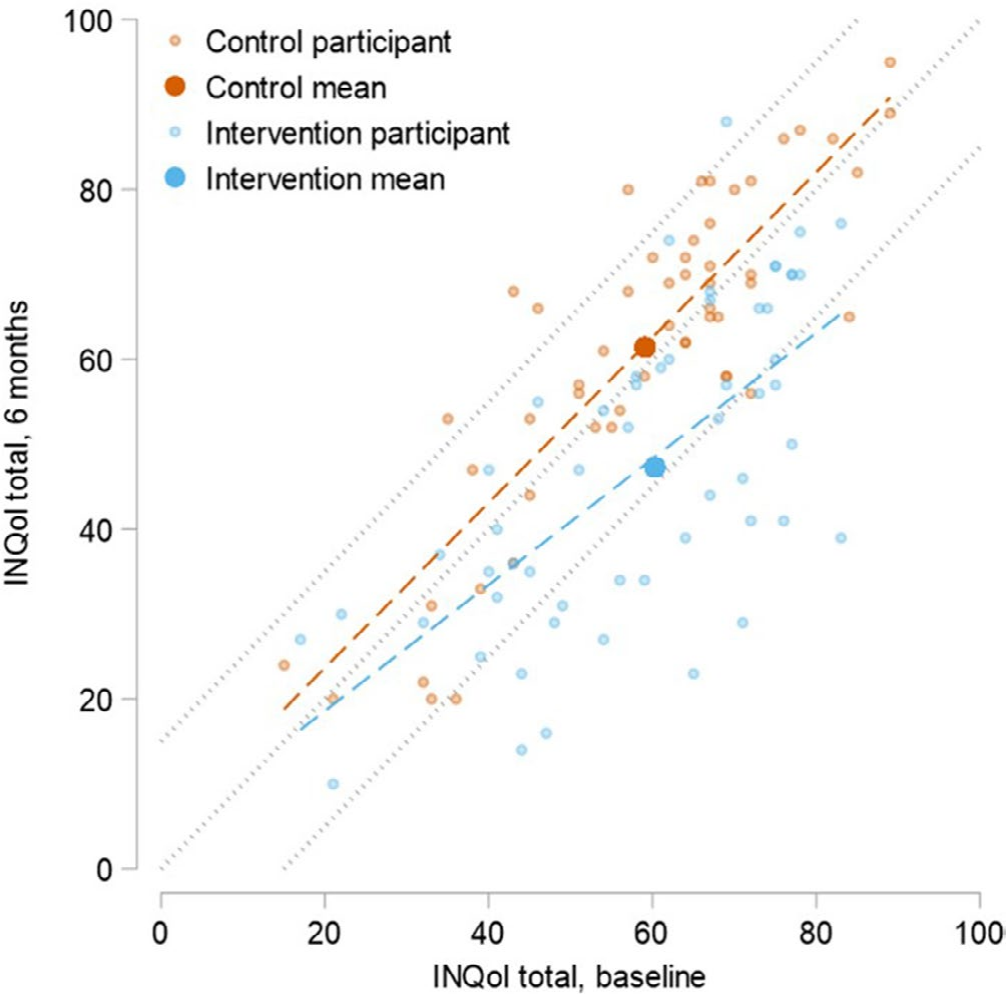
Individual participant change (baseline and 6 months) in QoL scores in both arms of the intervention plotted against reliable change parameters. INQoL = Individualized Neuromuscular Quality of Life Questionnaire.

Data for the INQoL total score at 6 months versus baseline is displayed in Figure 3 and Table 2 (*n* = 109). Using an estimate of reliable change, defined as an improvement greater than 1.96, the measurement error, one‐third (33.9%) of those receiving ACT+SMC experienced reliable improvements in QoL at 6 months, compared to only 6% of those in the SMC group (Table 2). Greater improvements were generally seen for those with higher INQoL scores at baseline (i.e., worse QoL). Similarly, less than 2% of those in the ACT+SMC group showed a reliable deterioration in QoL at 6 months, a lower proportion than those in the SMC group (7%). Most participants showed no reliable change, either improvement or deterioration. However, in the SMC group, 86.8% reported no reliable change compared to 64.3% in the ACT+SMC group.

**TABLE 2:** Counts and percentages of participants in each arm demonstrating reliable change in QoL (INQoL Life Area domain).

	SMC Count %		SMC + ACT Count. %		Total in analysis Count. %	
Improved	3	5.70%	19	33.90%	22	20.20%
No change	46	86.80%	36	64.30%	82	75.20%
Worsened	4	7.60%	1	1.80%	5	4.60%
Total	53		56		109	

## 4 Discussion

The short‐term moderate improvements in QoL in response to ACT that were seen at 9 weeks [7] are maintained at 6 months. Over one‐third of participants experienced a long‐term reliable improvement in QoL following ACT, compared to a small proportion of participants in the SMC alone arm, with reliable deterioration extremely rare in the ACT+SMC arm. Improvements in most secondary outcomes—most notably mood—were also maintained to 6 months. Together this suggests that this brief ACT‐based guided self‐help intervention leads to a beneficial impact on QoL and other key outcomes that is maintained in the longer term, with a considerable proportion of participants experiencing improvements in QoL.

As might be expected with a psychological intervention [6], the ACT intervention did not appear to improve physical impairment. The significant improvement noted on the downstream process of symptom interference at 9 weeks was reduced at 6 months, with group differences no longer significant.

Psychological flexibility, the theoretical treatment mechanism or treatment process for ACT, showed the greatest pattern of difference from that observed at 9 weeks [7]. Effect sizes in favor of the ACT intervention appeared to increase at 6 months for the openness (AAQ‐II) and awareness (MAAS), with a significant difference between groups. In contrast, the widening confidence intervals on the measure of engagement (CAQ) meant that the previous significant differences between groups disappeared. Overall, the broad pattern of small effects on psychological flexibility is consistent with a large meta‐analytic review of all trials of ACT, which observed similar effect sizes on outcomes measuring psychological flexibility [27]. The small changes on measures of theorized treatment process could be explained in several ways. First, the intervention was relatively brief and low intensity, which is commensurate with a small change in psychological flexibility, and differences in the significance of change across the three aspects may be because certain aspects were more effectively targeted by intervention techniques. Second, limited change could be caused by measurement error or imprecision implicit in psychological flexibility questionnaires [28, 29]. Third, perhaps the intervention works largely via nonspecific factors that are common to psychological interventions [30], such as the beneficial impact of an empathetic, supportive relationship.

The majority of participants were satisfied with the ACT intervention. There was little evidence of the intervention leading to harm, with minimal reliable deteriorations in scores evident in the ACT+SMC group, and fewer than in the SMC alone group. This, combined with the few serious adverse events noted in the short‐term outcome study [7], presents a positive evaluation of the safety of the ACT intervention.

These results suggest that psychological intervention even in brief formats, here ACT‐based guided self‐help, can improve QoL in MD. We included participants with some evidence of distress, suggesting that those who are experiencing some anxiety or low mood can benefit from this approach. Nonetheless, only around a third of participants demonstrated improvement to the extent of reliable change, a number that closely aligns with the proportions reporting a general perception of benefit from the intervention (PGIC). The residual group of non‐responders suggests that a range of interventions for improving QoL or mental health may be required to meet differing needs across the population of individuals with MD. Encouragingly, there is increasing interest in tailoring and trialing a range of psychological interventions for MD [31], and evidence that those with MD who experience severe fatigue can benefit from cognitive behavior therapy [32, 33]. In a qualitative study conducted with our participants [34], several advised that the intervention was experienced as brief and suggested that additional check‐in session would be beneficial. While the present study suggests that effects of the intervention are maintained irrespective, it remains possible that an additional booster session would be of value.

Several limitations are worth considering in conjunction with our results. First, although outcome completion rates remained high at 6 months, fewer participants completed outcome assessment at this timepoint than at 9 weeks [7]. This could have introduced a selection bias into the results. Our self‐selecting cohort may have comprised those with more or less positive experiences of the trial, the intervention or of clinical care at the recruiting site. Similarly, the design of the study did not include a placebo control, meaning a proportion of the observed effects could be explained by expectancy bias or the non‐specific aspects of the therapeutic relationship. Finally, we did not record use of other therapy sources across the period of study.

### 4.1 Conclusions

Beneficial impacts of brief ACT‐based guided self‐help on QoL are maintained in the longer term, with moderate effects at the group level. Improvements in mood are also maintained to 6 months. Over a third of those receiving ACT showed reliable improvements, with a similar proportion reporting a global perception of improvement in outcome. There were minimal reliable deteriorations in those randomized to ACT and fewer than in the SMC alone arm, which further supports the safety of the intervention.

## Author Contributions

**Christopher D. Graham:** conceptualization, investigation, funding acquisition, writing – original draft, methodology, validation, writing – review and editing, resources, supervision. **Michael Rose:** conceptualization, investigation, funding acquisition, writing – original draft, writing – review and editing, resources, project administration. **Victoria Edwards:** project administration, resources, data curation, investigation, writing – review and editing, methodology. **Chiara Vari:** investigation, methodology, writing – review and editing, project administration. **Nicola O'Connell:** investigation, methodology, writing – review and editing, project administration. **Emma Taylor:** investigation, writing – review and editing, methodology, project administration. **Lance M. McCracken:** conceptualization, funding acquisition, investigation, methodology, writing – review and editing, supervision. **Aleksander Radunovic:** conceptualization, investigation, funding acquisition, writing – review and editing, methodology, project administration, resources. **Wojtek Rakowicz:** conceptualization, investigation, funding acquisition, methodology, writing – review and editing, project administration, resources. **Sam Norton:** conceptualization, funding acquisition, investigation, writing – review and editing, writing – original draft, methodology, visualization, software, formal analysis, data curation, resources. **Trudie Chalder:** conceptualization, investigation, funding acquisition, writing – original draft, methodology, validation, visualization, writing – review and editing, project administration, data curation, supervision, resources.

## Conflicts of Interest

Authors report sources of financial support in the Disclosure Statement. Beyond these declarations authors report no conflicts of interest.

## Disclosure

Trudie Chalder acknowledges the financial support of the Department of Health via the National Institute for Health Research (NIHR) Specialist Biomedical Research Centre for Mental Health award to the South London and Maudsley NHS Foundation Trust (SLaM) and the Institute of Psychiatry at King's College London. The views expressed in this article are those of the authors and not necessarily those of the NHS, the NIHR, or the Department of Health and Social Care. Michael Rose, Christopher D. Graham, Nicola O'Connell, Chiara Vari, Victoria Edwards, Emma Taylor, Lance M. McCracken, Aleksander Radunovic, Wojtek Rakowicz, and Sam Norton have no financial disclosures to make.

## Ethics Statement

We confirm that we have read the Journal's position on issues involved in ethical publication and affirm that this report is consistent with those guidelines.

## Supporting information


Data S1

## Data Availability

The data that support the findings of this study are available from the corresponding author upon reasonable request.
