# Supplementary material for: Long‐Term Outcomes From a Randomized Controlled Trial of Acceptance and Commitment Therapy (ACT) Compared to Standard Medical Care for Improving Quality of Life in Muscle Disorders
Source: Muscle Nerve. 2024 Dec 29;71(3):398–405. doi: 10.1002/mus.28322 (PMC11799407; doi:10.1002/mus.28322)
Supplement: Supplementary file 1 — Data S1 [file MUS-71-398-s001.docx]

**Supplementary Table 1. Observed means and adjusted mean differences. Adjusted mean differences are estimated using a mixed effects model adjusting for baseline level of the outcome and recruiting site using the intention to treat sample (N=148).**

|  |  | SMC | | | ACT+SMC | | | Adjusted mean difference | | | | | | |
| --- | --- | --- | --- | --- | --- | --- | --- | --- | --- | --- | --- | --- | --- | --- |
|  | Time | N | Mean | SD | N | Mean | SD | Diff | SE | z | p | 95%ll | 95%ul | SMD |
| INQoL Total | Baseline | 75 | 58.69 | 16.94 | 73 | 60.92 | 17.91 |  |  |  |  |  |  |  |
|  | 3 weeks | 74 | 60.18 | 19.13 | 72 | 54.19 | 18.29 | -7.81 | 1.64 | -4.76 | 0.000 | -11.02 | -4.59 | -0.45 |
|  | 6 weeks | 70 | 58.43 | 19.16 | 66 | 50.30 | 19.37 | -9.32 | 1.89 | -4.92 | 0.000 | -13.04 | -5.61 | -0.54 |
|  | 9 weeks | 72 | 58.86 | 19.57 | 66 | 47.77 | 21.18 | -12.19 | 2.16 | -5.63 | 0.000 | -16.43 | -7.95 | -0.71 |
|  | 6 months | 53 | 61.43 | 18.95 | 56 | 47.29 | 18.19 | -12.25 | 2.11 | -5.82 | 0.000 | -16.38 | -8.12 | -0.71 |
| INQoL Activities | Baseline | 75 | 70.63 | 21.41 | 73 | 68.74 | 21.37 |  |  |  |  |  |  |  |
|  | 3 weeks | 74 | 69.54 | 21.69 | 73 | 63.33 | 21.47 | -4.92 | 2.01 | -2.45 | 0.014 | -8.85 | -0.98 | -0.23 |
|  | 6 weeks | 71 | 68.66 | 23.20 | 65 | 59.66 | 21.64 | -6.97 | 2.18 | -3.19 | 0.001 | -11.25 | -2.69 | -0.33 |
|  | 9 weeks | 72 | 68.89 | 23.95 | 66 | 58.30 | 23.12 | -8.34 | 2.59 | -3.22 | 0.001 | -13.42 | -3.25 | -0.40 |
|  | 6 months | 53 | 72.09 | 21.96 | 56 | 60.88 | 22.71 | -6.05 | 2.60 | -2.33 | 0.020 | -11.14 | -0.97 | -0.29 |
| INQoL Independence | Baseline | 75 | 59.93 | 23.77 | 73 | 61.71 | 24.10 |  |  |  |  |  |  |  |
|  | 3 weeks | 74 | 61.72 | 24.59 | 73 | 56.15 | 24.59 | -7.49 | 2.25 | -3.33 | 0.001 | -11.90 | -3.08 | -0.32 |
|  | 6 weeks | 71 | 62.10 | 22.98 | 66 | 54.58 | 22.24 | -8.62 | 2.23 | -3.86 | 0.000 | -13.00 | -4.25 | -0.37 |
|  | 9 weeks | 72 | 62.71 | 23.87 | 66 | 53.21 | 24.37 | -10.22 | 2.47 | -4.13 | 0.000 | -15.06 | -5.37 | -0.44 |
|  | 6 months | 53 | 62.79 | 22.71 | 56 | 53.39 | 23.43 | -7.72 | 2.62 | -2.94 | 0.003 | -12.86 | -2.57 | -0.33 |
| INQoL Social Relationships | Baseline | 72 | 41.11 | 20.89 | 70 | 44.33 | 20.54 |  |  |  |  |  |  |  |
|  | 3 weeks | 70 | 45.97 | 20.73 | 73 | 40.77 | 19.43 | -6.75 | 2.29 | -2.95 | 0.003 | -11.24 | -2.27 | -0.33 |
|  | 6 weeks | 68 | 44.93 | 22.33 | 65 | 36.97 | 21.81 | -9.18 | 2.60 | -3.53 | 0.000 | -14.28 | -4.07 | -0.45 |
|  | 9 weeks | 69 | 45.86 | 20.86 | 65 | 36.09 | 22.23 | -10.58 | 2.82 | -3.76 | 0.000 | -16.10 | -5.06 | -0.52 |
|  | 6 months | 53 | 49.87 | 22.01 | 55 | 32.42 | 21.28 | -16.30 | 3.01 | -5.41 | 0.000 | -22.20 | -10.40 | -0.80 |
| INQoL Emotions | Baseline | 75 | 61.59 | 19.83 | 73 | 62.77 | 20.38 |  |  |  |  |  |  |  |
|  | 3 weeks | 74 | 61.11 | 22.20 | 72 | 53.96 | 22.69 | -7.87 | 2.37 | -3.33 | 0.001 | -12.51 | -3.24 | -0.40 |
|  | 6 weeks | 70 | 57.30 | 23.26 | 66 | 44.26 | 22.78 | -12.44 | 2.87 | -4.34 | 0.000 | -18.07 | -6.82 | -0.62 |
|  | 9 weeks | 72 | 56.40 | 22.90 | 66 | 39.76 | 25.67 | -16.49 | 3.22 | -5.12 | 0.000 | -22.80 | -10.18 | -0.83 |
|  | 6 months | 53 | 62.08 | 23.13 | 56 | 40.04 | 21.17 | -19.55 | 3.24 | -6.03 | 0.000 | -25.90 | -13.20 | -0.98 |
| INQoL Body Image | Baseline | 75 | 59.65 | 24.94 | 73 | 66.04 | 22.49 |  |  |  |  |  |  |  |
|  | 3 weeks | 74 | 60.64 | 22.95 | 72 | 58.11 | 23.59 | -6.83 | 2.60 | -2.63 | 0.009 | -11.92 | -1.73 | -0.28 |
|  | 6 weeks | 71 | 59.41 | 23.88 | 66 | 55.26 | 23.25 | -7.31 | 2.66 | -2.75 | 0.006 | -12.53 | -2.10 | -0.31 |
|  | 9 weeks | 72 | 59.63 | 23.15 | 66 | 50.58 | 25.72 | -12.78 | 2.98 | -4.29 | 0.000 | -18.62 | -6.94 | -0.53 |
|  | 6 months | 53 | 60.64 | 24.03 | 56 | 49.77 | 22.53 | -10.22 | 3.05 | -3.35 | 0.001 | -16.19 | -4.24 | -0.43 |
| INQoL Weakness | Baseline | 74 | 74.66 | 20.66 | 73 | 74.26 | 20.58 |  |  |  |  |  |  |  |
|  | 3 weeks | 74 | 72.46 | 22.93 | 73 | 67.99 | 20.55 | -4.27 | 2.25 | -1.90 | 0.058 | -8.68 | 0.14 | -0.20 |
|  | 6 weeks | 71 | 72.93 | 21.47 | 66 | 66.77 | 20.46 | -5.00 | 2.35 | -2.13 | 0.033 | -9.60 | -0.40 | -0.24 |
|  | 9 weeks | 72 | 74.93 | 22.03 | 66 | 66.97 | 21.66 | -6.83 | 2.52 | -2.71 | 0.007 | -11.78 | -1.89 | -0.33 |
|  | 6 months | 53 | 74.32 | 21.77 | 56 | 65.88 | 23.00 | -4.93 | 3.26 | -1.51 | 0.130 | -11.33 | 1.46 | -0.24 |
| INQoL Pain | Baseline | 75 | 46.64 | 28.47 | 73 | 44.18 | 31.03 |  |  |  |  |  |  |  |
|  | 3 weeks | 74 | 44.89 | 28.72 | 73 | 36.45 | 30.51 | -6.87 | 3.07 | -2.24 | 0.025 | -12.89 | -0.85 | -0.23 |
|  | 6 weeks | 71 | 46.07 | 28.32 | 66 | 37.20 | 30.02 | -5.63 | 3.10 | -1.82 | 0.069 | -11.69 | 0.44 | -0.19 |
|  | 9 weeks | 72 | 44.93 | 30.27 | 66 | 34.14 | 30.29 | -8.69 | 3.25 | -2.67 | 0.008 | -15.06 | -2.31 | -0.30 |
|  | 6 months | 53 | 47.89 | 31.30 | 56 | 37.00 | 30.30 | -7.46 | 3.70 | -2.02 | 0.044 | -14.72 | -0.20 | -0.25 |
| INQoL Tiredness | Baseline | 75 | 55.36 | 27.64 | 73 | 60.82 | 27.03 |  |  |  |  |  |  |  |
|  | 3 weeks | 74 | 57.80 | 25.46 | 73 | 56.52 | 23.69 | -4.76 | 2.98 | -1.60 | 0.110 | -10.60 | 1.07 | -0.18 |
|  | 6 weeks | 71 | 60.03 | 24.44 | 66 | 51.21 | 27.55 | -10.63 | 3.18 | -3.35 | 0.001 | -16.86 | -4.41 | -0.40 |
|  | 9 weeks | 72 | 60.49 | 23.95 | 66 | 52.23 | 27.75 | -10.73 | 3.54 | -3.03 | 0.002 | -17.67 | -3.79 | -0.40 |
|  | 6 months | 53 | 64.62 | 25.09 | 56 | 49.16 | 27.02 | -14.31 | 3.81 | -3.76 | 0.000 | -21.78 | -6.85 | -0.53 |
| Work & Social Adjustment Scale | Baseline | 75 | 38.20 | 15.38 | 73 | 35.52 | 15.64 |  |  |  |  |  |  |  |
|  | 3 weeks | 73 | 35.90 | 16.04 | 72 | 31.83 | 14.38 | -1.91 | 1.50 | -1.27 | 0.204 | -4.85 | 1.03 | -0.13 |
|  | 6 weeks | 68 | 35.52 | 15.26 | 64 | 30.66 | 15.02 | -2.48 | 1.46 | -1.71 | 0.088 | -5.34 | 0.37 | -0.16 |
|  | 9 weeks | 70 | 36.17 | 15.51 | 64 | 30.46 | 16.04 | -3.80 | 1.51 | -2.52 | 0.012 | -6.76 | -0.84 | -0.25 |
|  | 6 months | 53 | 36.69 | 14.62 | 56 | 30.94 | 14.77 | -2.25 | 1.59 | -1.42 | 0.155 | -5.36 | 0.85 | -0.15 |
| HADS Anxiety | Baseline | 75 | 9.48 | 3.39 | 73 | 9.93 | 3.95 |  |  |  |  |  |  |  |
|  | 3 weeks | 74 | 9.59 | 3.34 | 73 | 9.04 | 3.94 | -0.85 | 0.43 | -1.98 | 0.048 | -1.69 | -0.01 | -0.24 |
|  | 6 weeks | 70 | 9.10 | 3.32 | 66 | 7.08 | 3.59 | -2.27 | 0.46 | -4.95 | 0.000 | -3.17 | -1.37 | -0.63 |
|  | 9 weeks | 72 | 8.62 | 3.33 | 66 | 6.98 | 4.43 | -2.12 | 0.56 | -3.80 | 0.000 | -3.21 | -1.02 | -0.59 |
|  | 6 months | 53 | 9.21 | 3.88 | 56 | 7.05 | 3.58 | -2.10 | 0.60 | -3.47 | 0.001 | -3.28 | -0.91 | -0.58 |
| HADS Depression | Baseline | 75 | 8.93 | 3.13 | 73 | 9.99 | 3.08 |  |  |  |  |  |  |  |
|  | 3 weeks | 74 | 8.83 | 3.05 | 73 | 8.71 | 3.38 | -0.92 | 0.38 | -2.40 | 0.016 | -1.67 | -0.17 | -0.30 |
|  | 6 weeks | 70 | 8.55 | 2.85 | 66 | 7.70 | 3.32 | -1.58 | 0.39 | -4.04 | 0.000 | -2.35 | -0.81 | -0.52 |
|  | 9 weeks | 72 | 8.68 | 3.17 | 66 | 7.71 | 3.93 | -1.78 | 0.47 | -3.83 | 0.000 | -2.69 | -0.87 | -0.58 |
|  | 6 months | 53 | 8.79 | 2.98 | 56 | 7.55 | 3.42 | -1.69 | 0.49 | -3.42 | 0.001 | -2.65 | -0.72 | -0.55 |
| HAQ Disability | Baseline | 75 | 1.84 | 0.82 | 72 | 1.72 | 0.72 |  |  |  |  |  |  |  |
|  | 3 weeks | 73 | 1.80 | 0.83 | 72 | 1.70 | 0.69 | 0.02 | 0.04 | 0.57 | 0.571 | -0.06 | 0.10 | 0.03 |
|  | 6 weeks | 69 | 1.88 | 0.79 | 64 | 1.70 | 0.68 | -0.04 | 0.04 | -1.09 | 0.274 | -0.12 | 0.03 | -0.06 |
|  | 9 weeks | 72 | 1.88 | 0.81 | 64 | 1.72 | 0.72 | -0.05 | 0.04 | -1.01 | 0.314 | -0.13 | 0.04 | -0.06 |
|  | 6 months | 53 | 1.92 | 0.74 | 56 | 1.74 | 0.75 | 0.00 | 0.05 | 0.06 | 0.954 | -0.10 | 0.11 | 0.00 |
| Acceptance and Action Question | Baseline | 74 | 27.72 | 11.73 | 73 | 28.50 | 12.74 |  |  |  |  |  |  |  |
|  | 3 weeks | 73 | 26.24 | 11.99 | 72 | 27.60 | 11.83 | 0.98 | 1.23 | 0.79 | 0.428 | -1.44 | 3.40 | 0.08 |
|  | 6 weeks | 69 | 26.70 | 11.96 | 64 | 24.09 | 10.77 | -2.91 | 1.34 | -2.17 | 0.030 | -5.54 | -0.29 | -0.25 |
|  | 9 weeks | 71 | 25.84 | 10.87 | 64 | 23.40 | 12.62 | -2.63 | 1.52 | -1.73 | 0.083 | -5.60 | 0.34 | -0.22 |
|  | 6 months | 53 | 27.77 | 11.98 | 56 | 21.29 | 10.66 | -5.71 | 1.82 | -3.13 | 0.002 | -9.29 | -2.14 | -0.48 |
| Mindful Attention Awareness Sc | Baseline | 75 | 4.33 | 0.82 | 73 | 4.08 | 0.98 |  |  |  |  |  |  |  |
|  | 3 weeks | 73 | 4.24 | 1.00 | 72 | 4.14 | 1.04 | 0.14 | 0.11 | 1.32 | 0.187 | -0.07 | 0.35 | -0.16 |
|  | 6 weeks | 69 | 4.41 | 0.89 | 64 | 4.33 | 1.05 | 0.17 | 0.10 | 1.63 | 0.103 | -0.03 | 0.37 | -0.19 |
|  | 9 weeks | 71 | 4.46 | 0.93 | 64 | 4.44 | 1.15 | 0.23 | 0.12 | 1.88 | 0.061 | -0.01 | 0.46 | -0.25 |
|  | 6 months | 53 | 4.34 | 1.03 | 54 | 4.61 | 1.01 | 0.47 | 0.14 | 3.25 | 0.001 | 0.18 | 0.75 | -0.51 |
| Committed Action Questionnaire | Baseline | 75 | 28.41 | 7.79 | 73 | 28.17 | 7.70 |  |  |  |  |  |  |  |
|  | 3 weeks | 72 | 28.19 | 8.17 | 72 | 29.73 | 6.83 | 1.54 | 0.82 | 1.89 | 0.059 | -0.06 | 3.14 | -0.21 |
|  | 6 weeks | 68 | 29.09 | 7.94 | 63 | 30.75 | 7.53 | 2.18 | 0.97 | 2.25 | 0.025 | 0.28 | 4.07 | -0.30 |
|  | 9 weeks | 71 | 29.43 | 8.87 | 64 | 31.47 | 7.82 | 2.52 | 1.13 | 2.24 | 0.025 | 0.32 | 4.73 | -0.35 |
|  | 6 months | 53 | 29.67 | 9.05 | 54 | 31.48 | 8.28 | 2.23 | 1.30 | 1.72 | 0.086 | -0.31 | 4.77 | -0.31 |
| IBM Functional Rating Scale | Baseline | 21 | 22.08 | 7.76 | 13 | 24.46 | 7.25 |  |  |  |  |  |  |  |
|  | 3 weeks | 19 | 21.26 | 7.53 | 15 | 22.80 | 6.64 | -0.66 | 0.74 | -0.89 | 0.375 | -2.11 | 0.80 | -0.09 |
|  | 6 weeks | 19 | 20.79 | 7.90 | 13 | 22.38 | 7.01 | -1.46 | 0.79 | -1.84 | 0.066 | -3.01 | 0.09 | -0.19 |
|  | 9 weeks | 20 | 21.35 | 8.16 | 13 | 23.38 | 6.36 | -0.98 | 0.89 | -1.10 | 0.273 | -2.72 | 0.77 | -0.13 |
|  | 6 months | 16 | 18.06 | 7.82 | 12 | 20.33 | 7.62 | -0.51 | 0.78 | -0.66 | 0.509 | -2.03 | 1.01 | -0.07 |

**Supplementary Table 2. Participant scores on Patient Global Impression of Change (PGIC) and treatment satisfaction measures**

|  |  | SMC (n=53) | ACT+SMC (n=56) | Treatment effect |
| --- | --- | --- | --- | --- |
| PGIC | Very much worse | 1 ( 2%) | 1 ( 2%) | OR=4.0; 95%CI=1.9 to 8.5); p<.001 |
|  | Much worse | 6 (11%) | 2 ( 4%) |  |
|  | A little worse | 26 (49%) | 16 (29%) |  |
|  | About the same | 17 (32%) | 19 (34%) |  |
|  | A little better | 2 ( 4%) | 8 (14%) |  |
|  | Much better | 1 ( 2%) | 8 (14%) |  |
|  | Very much better | 0 ( 0%) | 2 ( 4%) |  |
| Satisfaction | Very dissatisfied | 3 ( 6%) | 0 ( 0%) | OR=8.9; 95%CI=3.6 to 22.1; p<.001 |
|  | Slightly dissatisfied | 1 ( 2%) | 1 ( 2%) |  |
|  | Neither | 37 (70%) | 9 (16%) |  |
|  | Slightly satisfied | 0 ( 0%) | 6 (11%) |  |
|  | Moderately satisfied | 4 ( 8%) | 16 (29%) |  |
|  | Very satisfied | 8 (15%) | 24 (43%) |  |
